# Editorial: Women in Science: Chemistry

**DOI:** 10.3389/fchem.2021.772775

**Published:** 2021-10-04

**Authors:** Elisabeth Lojou, Silvia Giordani, Svetlana Ivanova, Qin Li, Jennifer L. Schaefer

**Affiliations:** ^1^ CNRS UMR7281 Bioénergétique et Ingénierie des Protéines, Marseille, France; ^2^ Dublin City University, Dublin, Ireland; ^3^ Sevilla University, Sevilla, Spain; ^4^ Griffith University, Brisbane, QLD, Australia; ^5^ University of Notre Dame, Notre Dame, IN, United States

**Keywords:** catalysis, pharmaceutical chemistry, chemical biology, nanosciences, polymer, inorganic chemistry, analytical chemistry, energy

Following UNESCO’s official celebration of the International Day of Women and Girls in Science, Frontiers in Chemistry decided to run a special issue “*Women in Science: Chemistry*”. Indeed, although science and gender equality are essential to ensure sustainable development, less than 30% of researchers worldwide are women. Even more, female students in science disciplines with relatively higher female participation rates (e.g., biology and chemistry) still experience similar gender issues as students in male-dominated science disciplines (e.g., physics and mathematics) (Baird, 2018; Fisher et al., 2020). In some countries, academic institutes created dedicated committees to promote women in science. CNRS, the national center for research in France, created such a committee in 2001 because “Science cannot deprive itself of half of the talents” (Antoine Petit, CNRS president, 2016). Thanks to this effort, in 2016, 35% of researchers in public research in France were women (38% in chemistry) against an average of 14.6% in the EU. Nevertheless, at this rate, parity will be reached only in 2088 among permanent researchers, while it is approximately reached among PhDs. Jessica Lober Newsome for the United Kingdom Resource Centre for Women in Science, Engineering and Technology and the Royal Society of Chemistry noted that “men and women in United Kingdom PhD programs start their graduate work enthusiastic about the prospect of a career in the chemistry professoriate, but by the third year, the proportion of men planning careers in research had dropped from 61 to 59%. while for the women, the number had plummeted from 72% in the first year to 37% as they finish their studies” (Bertozzi, 2016).

Towards facilitating change, Frontiers in Chemistry had the ambition that gender equality may be promoted through this special issue. No doubt that female researchers have made significant contributions to the field of chemistry in all its diversity. As such, it is relevant to note that among the 42 contributions (33 original articles, two reviews, and seven mini-reviews) accepted for publication in this special issue, many different domains were covered: green and sustainable chemistry, medicinal and pharmaceutical chemistry, chemical biology, inorganic chemistry, physical chemistry, analytical chemistry, electrochemistry, nanosciences, supramolecular chemistry, polymer chemistry, and energy and catalysis. These works highlight the diversity of research performed across the entire breadth of the chemical sciences and present advances in theory, experiment, and methodology with applications to compelling problems. Although the background of the guest editors may bias this, it is interesting to note that 50% of the contributions belong to the bio-chem-green interface—reflecting a higher involvement of females in bio-related domains, which indicates that efforts are still needed to open all facets of chemistry equally to females.

Of note are the tremendous number of research works towards improved sustainability, either through green chemistry, addressing environmental issues, improved energy storage or generation devices, or catalysis. These efforts include a range of traditional chemical science subfields, including inorganic chemistry, polymer chemistry, physical chemistry, and electrochemistry, with the common end goal of improving the health of our shared planet. Within this objective, an investigation of the bio-chemical interface has also been put forward. Continued efforts on isolation and conversion of CO_2_ are needed as we seek to combat climate change. Klemm et al. report on facilitated transport membranes for CO_2_ separations , while Abou Hamdan et al. research a route for conversion of CO_2_ to a valuable feedstock. Contributions were also received in the areas of hydrogen generation (Pryce’s group) (Cullen et al.), and carbon monoxide oxidation (Couvret et al.). The advancement of electrochemical energy generation and storage devices will ease a transition to greater use of electrical energy and away from fossil fuels. The whole range of available devices was covered by the received contributions, from flow cells, lithium batteries, and fuel cells. Five contributions, from corresponding authors Augustyn, Chen, Fullerton-Shirey, Marbella, and Ortiz-Vitoriano, report materials and systems for advanced batteries and capacitors (Boyd et al.; Chen et al.; Hestenes et al.; Ruiz de Larramendi and Ortiz-Vitoriano,; Jing et al.). Four contributions were received related to fuel cell materials. Ionomers suitable as membrane separators in proton exchange membrane fuel cells is an intensive research domain in which women take part (Farzin et al.; Wang et al.). Interestingly, Farzin et al. demonstrated that sulfonated Kraft lignin, a by-product of paper industries, may act as a suitable membrane , while Wang et al. reported iptycene-based copolymers as new components for proton exchange membranes. The use of enzymes as biocatalyst alternatives to platinum in fuel cells was also investigated in two contributions from Hitaishi et al.; Jacq-Bailly et al.. The oxygen reduction reaction, which is limiting fuel cell devices, was investigated from an experimental Hoque et al. to a theoretical point of view (Živković et al.). Green processes and sustainable feedstocks were the focus of four reports (Calla-Quispe et al.; Carlier and Hermans; Megías-Sayago et al.; Yu et al.). Carlier and Hermans investigated various carbon materials for cellobiose hydrolysis, a key process for biomass valorization, while (Megías-Sayago et al. studied the process of platform molecules conversion to biobased monomers. Lanthanide recovery was studied by Yu et al., while Calla-Quispe et al. explored the potentiality of ionic liquids as extracting agents of natural products). Also, towards sustainability and environmental health, wastewater treatment was the topic of two contributions from Zhang et al; Primo et al.. Sensor development, including electrochemical-based sensors, was detailed in view of *in situ* analytical monitoring and bio-inspired synthesis. Sfragano et al. provided a mini-review dedicated to electrochemical platforms for green analytical sensing . Mousty and colleagues described the electrochemical properties of NiAl-layered double hydroxide nanoparticles for biosensing applications (Prevot et al.), while Truta et al.reviewed the combined use of nanomaterials and biorecognition for drug sensing.

Ten great contributions (seven original research articles, one review, and two mini-reviews) were received in the medicinal and pharmaceutical chemistry, chemical biology, and biosensing domains. Chemistry can play an important role in the battle against cancer and contribute to advancing cancer diagnosis and treatment. Addressing tumor cells without affecting healthy tissue is a major challenge in cancer therapy. Phototherapy, immunotherapy, and selected targeted chemotherapy are very promising tools for cancer treatment. Bartelmess et al. reported the use of carbon nano-onions (CNOs) as molecular shuttles for photodynamic therapeutics, boosting the modulation of the photosensitizer . Hoppenz et al. give an overview of the most common peptide receptors overexpressed on cancer cells, highlight the benefits of peptides as tumor homing agents and outline the specific requirements of peptide-drug conjugates (Arpicco et al.; Hoppenz et al.). Another receptor that is often overexpressed in many tumor cells is hyaluronic acid (HA). Arpicco et al. examine the correlation of HA molecular weight with its targetability properties in the formulation of a carbon nanotube drug delivery conjugate for tumor-targeted delivery of the anticancer agent doxorubicin (Arpicco et al.; Hoppenz et al.). Hu and Li mini-review highlights the importance of enhancing cancer immunotherapy by developing safe and effective adjuvants (). Two research articles highlight the crucial role chemistry plays in the synthesis and effective delivery of luminescent probes for cellular imaging. Finn et al.; Magno et al. engineered porous carbon microparticles for the transport and intracellular delivery of a fluorescent dye . Finn et al. carefully design highly luminescent metal complexes that self-assemble to form nanoaggregates with good membrane permeability, which can be used as imaging probes . Two contributions were also received on the development of new antimicrobials and antibiotics (Le Brun et al.; Yim et al.) and one mini-review on cosmeceutical peptides in the framework of sustainable wellness economy (Errante et al.). Real-time observations by Tof-SIMS at a submicropore-confined liquid/vacuum interface were also reported by Liu et al. in view of the analysis of the structure of water clusters, a key point to understand processes in biology . Finally, Sapountzi et al. developed a core-shell nanomaterial for glucose biosensing .

Advanced preparation methods for functional materials and thin films are also present in this special issue. (Xu et al.) summarized the existing methods for conjugated polymer preparation in a mini-review, and discussed their potential to form hierarchical assemblies with tuning optical, electrical or mechanical properties. The versatility of the assembly formation contrasts with the necessity for better solid-state control of the final film are well reflected in this contribution. (Vilà and Walcarius) used a combination of electrochemically-assisted self-assembly and the Huisgen cycloaddition reaction to generate nanostructured thin films with electrochromic properties. This combination can be used to obtain even more complex structures, including metal centers of different nature. Enhancing the upconversion efficiency of lanthanide nanoparticles (Li et al.) and tuning magnetic properties of Fe_6_Dy_3_ rings (Kühne et al.) were reported. The functionalization of surfaces *via* diazonium grafting was reviewed by Mattiuzzi et al.). Two different strategies have been contrasted, the use of ionic liquids as solvents and layer growth limiting agent and calix (Klemm et al.) arene strategy with reactive appending arms and controlled composition. Whatever the strategy, the resulting monolayers proposed a real alternative to the classical SAMs of thiol derivatives and can be applied for biosensing and electrocatalysis. The potential of the solvothermal synthesis for di- and tri-nuclear V(III) and Cr(III) complexes of dipyridyltriazoles were explored by (Rinck et al.). It was found that the synthesis conditions facilitate ligand rearrangements in a new bridging topology which is the first example, up to date, of a ferromagnetic coupling in Cr(III) bridged dimer with ligands other than hydroxyls. Finally, BODIPY molecules were examined as molecular switches for digital device applications by (Trifoi et al.).

As guest editors, we would like to thank our contributors for their excellent dedication, as well as the reviewers for their constructive work. We sincerely thank the editorial staff of this issue for this superb idea and valuable assistance throughout the editing process. Last and not least, we would like this issue to send a clear message to every young female student in chemical sciences to keep going and pursue their dreams in the fantastic chemical research world.
